# Genomic insights into host and parasite interactions during intracellular infection by *Toxoplasma gondii*

**DOI:** 10.1371/journal.pone.0275226

**Published:** 2022-09-30

**Authors:** Netha Ulahannan, Ronald Cutler, Reanna Doña-Termine, Claudia A. Simões-Pires, N. Ari Wijetunga, Matthew McKnight Croken, Andrew D. Johnston, Yu Kong, Shahina B. Maqbool, Masako Suzuki, John M. Greally

**Affiliations:** 1 Department of Genetics, Albert Einstein College of Medicine, Bronx, NY, United States of America; 2 Department of Microbiology and Immunology, Albert Einstein College of Medicine, Bronx, NY, United States of America; HudsonAlpha Institute for Biotechnology, UNITED STATES

## Abstract

To gain insights into the molecular interactions of an intracellular pathogen and its host cell, we studied the gene expression and chromatin states of human fibroblasts infected with the Apicomplexan parasite *Toxoplasma gondii*. We show a striking activation of host cell genes that regulate a number of cellular processes, some of which are protective of the host cell, others likely to be advantageous to the pathogen. The simultaneous capture of host and parasite genomic information allowed us to gain insights into the regulation of the *T*. *gondii* genome. We show how chromatin accessibility and transcriptional profiling together permit novel annotation of the parasite’s genome, including more accurate mapping of known genes and the identification of new genes and *cis*-regulatory elements. Motif analysis reveals not only the known *T*. *gondii* AP2 transcription factor-binding site but also a previously-undiscovered candidate TATA box-containing motif at one-quarter of promoters. By inferring the transcription factor and upstream cell signaling responses involved in the host cell, we can use genomic information to gain insights into *T*. *gondii’s* perturbation of host cell physiology. Our resulting model builds on previously-described human host cell signalling responses to *T*. *gondii* infection, linked to induction of specific transcription factors, some of which appear to be solely protective of the host cell, others of which appear to be co-opted by the pathogen to enhance its own survival.

## Introduction

The infection of a cell by an intracellular pathogen is accompanied by substantial changes in cellular homeostasis, reflected by activation and suppression of cell signaling pathways such as those regulating metabolism, cell division and cell death [1]. Some of these responses are protective for the host, while others represent processes co-opted by the pathogen to enhance its survival and spread. The evolutionary pressures on the host and pathogen are significant, to the point that interactions with pathogens have been proposed to represent the primary selective pressure in human evolution [2]. What we see today in host-pathogen interactions reflects a long history of selection acting on vital cellular processes.

We focus here on human cell infection by *Toxoplasma gondii*. This apicomplexan intracellular parasite is extremely successful, with an estimated one-quarter of humans chronically infected [3], causing symptomatic disease in immunocompromised individuals and following transmission to the developing fetus *in utero*. We now recognize many of the events occurring during *T*. *gondii* infection of host cells [4], giving insights into why this microorganism is such a successful infectious agent.

The application of genomic assays to cells infected by a eukaryotic pathogen yields rich but complex information. These assays can test transcription and its regulatory control from two organisms simultaneously, with some of the transcriptional and regulatory changes in the genome of the host cell likely to be a protective response to the infection, but other changes potentially advantageous for the pathogen, reflecting the molecular hijacking of host cell physiology. We also obtain a snapshot of the transcription and its regulation in the pathogen’s genome. The host loci at which regulatory changes are occurring are highly likely to include those mediating evolutionary selection, including host regulatory loci located distantly from genes in the non-coding majority of the genome, loci that would otherwise be difficult to identify and characterize using sequence information alone.

We have previously characterized transcriptional regulatory information in the *T*. *gondii* genome, showing that it is free of DNA methylation [5] and that post-translational modifications of *T*. *gondii* histones define *cis*-regulatory loci [6]. In this study, we perform transcriptional and chromatin profiling genome-wide in human fibroblasts prior to and after infection with *T*. *gondii*, revealing host responses, pathogen genomic regulation during the tachyzoite stage of *T*. *gondii* life cycle, and a new annotation of genes and *cis-*regulatory loci in the *T*. *gondii* genome, yielding insights into candidate transcription factor binding sites and the organization of *T*. *gondii’s* apicoplast genome. We also demonstrate how we can infer from observations of transcriptional perturbation insights into host cell signaling pathway alterations occurring in response to intracellular infection by this common pathogen.

## Results

### Host transcriptome responses to *Toxoplasma gondii* infection

**Fig 1:**
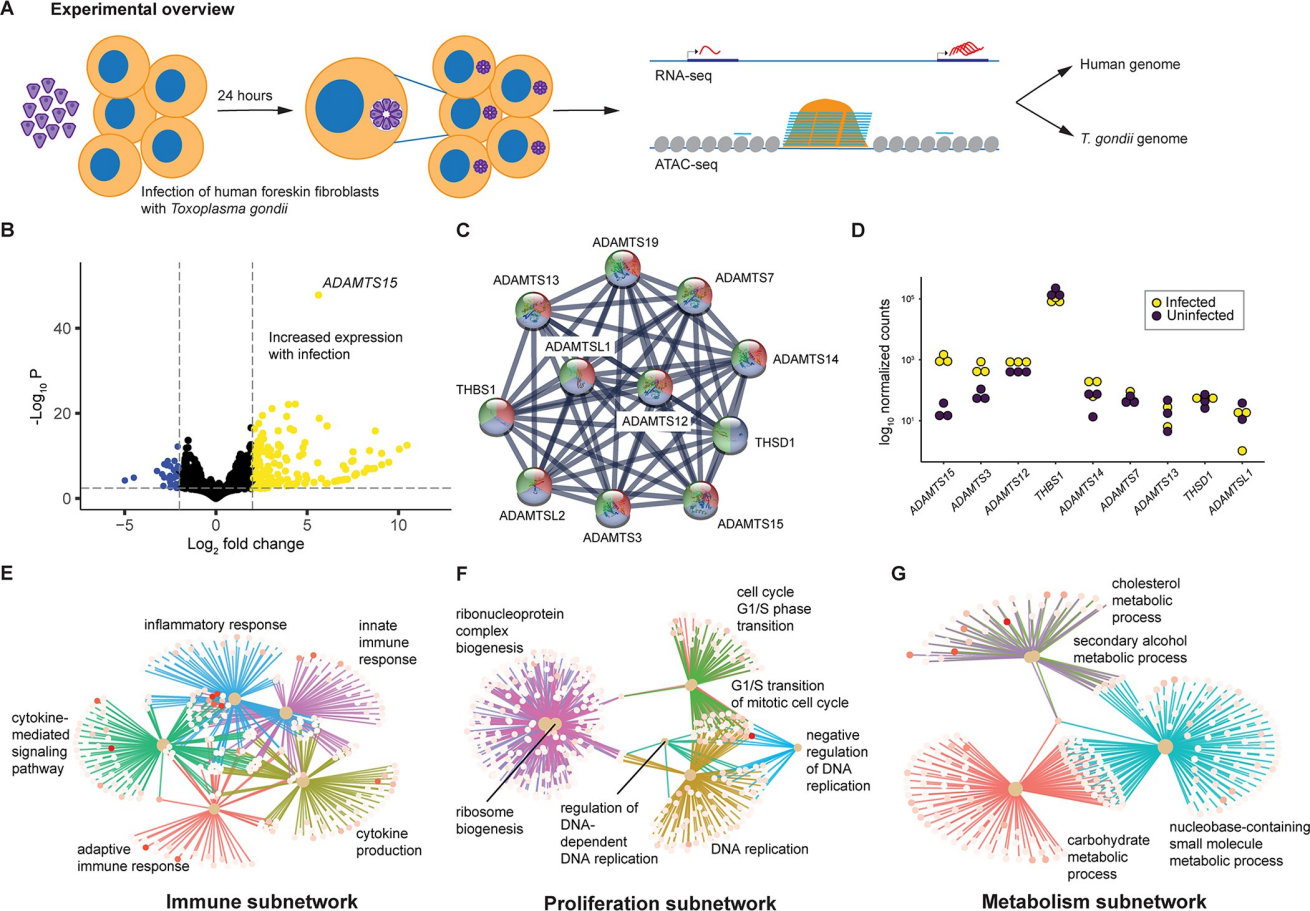
Project overview and host gene transcriptional response to *T*. *gondii* infection. We show in (A) the overview of the experiments performed, depicting the *in vitro* infection of human fibroblasts with *T*. *gondii*, with transcriptional (RNA-seq) and chromatin accessibility (ATAC-seq) studies performed before and 24 hours after infection, and alignment of the reads obtained to both the human and *T*. *gondii* genomes. The RNA-seq results in (B) show more human genes upregulated (yellow) than downregulated (blue) with infection, with *ADAMTS15* strongly upregulated. Using network analysis, we extracted genes interacting with *ADAMTS15* (C) and showed that other metalloproteases were upregulated with infection (D). (E-G) show the major groups of gene ontologies in the remaining upregulated genes.

We performed RNA sequencing (RNA-seq) of human foreskin fibroblasts (HFFs), before and 24 hours after *in vitro* infection with the *T*. *gondii* RH strain in the tachyzoite stage of the life cycle (**Fig 1A**). RNA-seq reads were aligned to a reference genome that combined both the human and *T*. *gondii* genomes simultaneously, an approach that shows more accurate representations in studies of infected host cells [7]. We showed the human host transcriptome to be characterized by hundreds of differentially expressed genes, with more genes increasing than decreasing their expression levels in infected cells (**Fig 1B, S1 Table**).

The single most markedly upregulated gene was *ADAMTS15*, a member of the A Disintegrin and Metalloprotease with Thrombospondin Motifs (ADAMTS) gene family. These metalloproteases function as regulators of extracellular matrix (ECM) composition, potentially enhancing cell migration by removing collagen barriers [8, 9]. By using ADAMTS15 to nucleate a protein-protein interaction analysis (**Fig 1C**), the RNA-seq data showed four of the nine genes encoding interacting proteins to have increased levels of expression in infected cells (*ADAMTS15*, *ADAMTS3*, *ADAMTS12*, *ADAMTS14*, **Fig 1D**). Matrix metalloproteases have been previously found to be overexpressed in response to *T*. *gondii* infection of astrocytes [10] and dendritic cells [11], potentially permitting the cells to become more mobile, and helping to disseminate the infection [11].

To gain insights into the properties of the broader group of upregulated genes, we performed a gene set enrichment analysis (GSEA), reducing the gene ontology (GO) terms by semantic similarity (**S1 Fig, S2 Table**). This analysis revealed three main subnetworks, one revealing a host cell immune response, a second the initiation of cell division, and a third reflecting activation of metabolic pathways (**Fig 1E–1G**). The immune response subnetwork included upregulation of the *NFKB1*, *ORAI1*, *and IL6* genes, indicative of activation of both the innate and adaptive immune responses [12–17]. These were accompanied by activation of genes related to cytokine production, including *TGFB*, *CXCL6*, *TNFSF15 and XCL1*, which prompt the recruitment of immune cells to the location of an ongoing infection [12, 18–20]. The cell division subnetwork involved activation of genes relating to ribosome biogenesis and G1/S phase transition, including *PRKDC*, *CASP2*, *and ATR* [21–23]. The third subnetwork is composed of genes involved in metabolic processes, with upregulation of genes such as *CYP27B1*, *DGAT2*, *FASN*, *and PCSK9* [24–27]. These genes are involved in cholesterol and carbohydrate metabolism, nutrients that the auxotrophic *T*. *gondii* has to acquire from the host cell [28].

### Host *cis*-regulatory changes in response to *Toxoplasma gondii* infection

**Fig 2:**
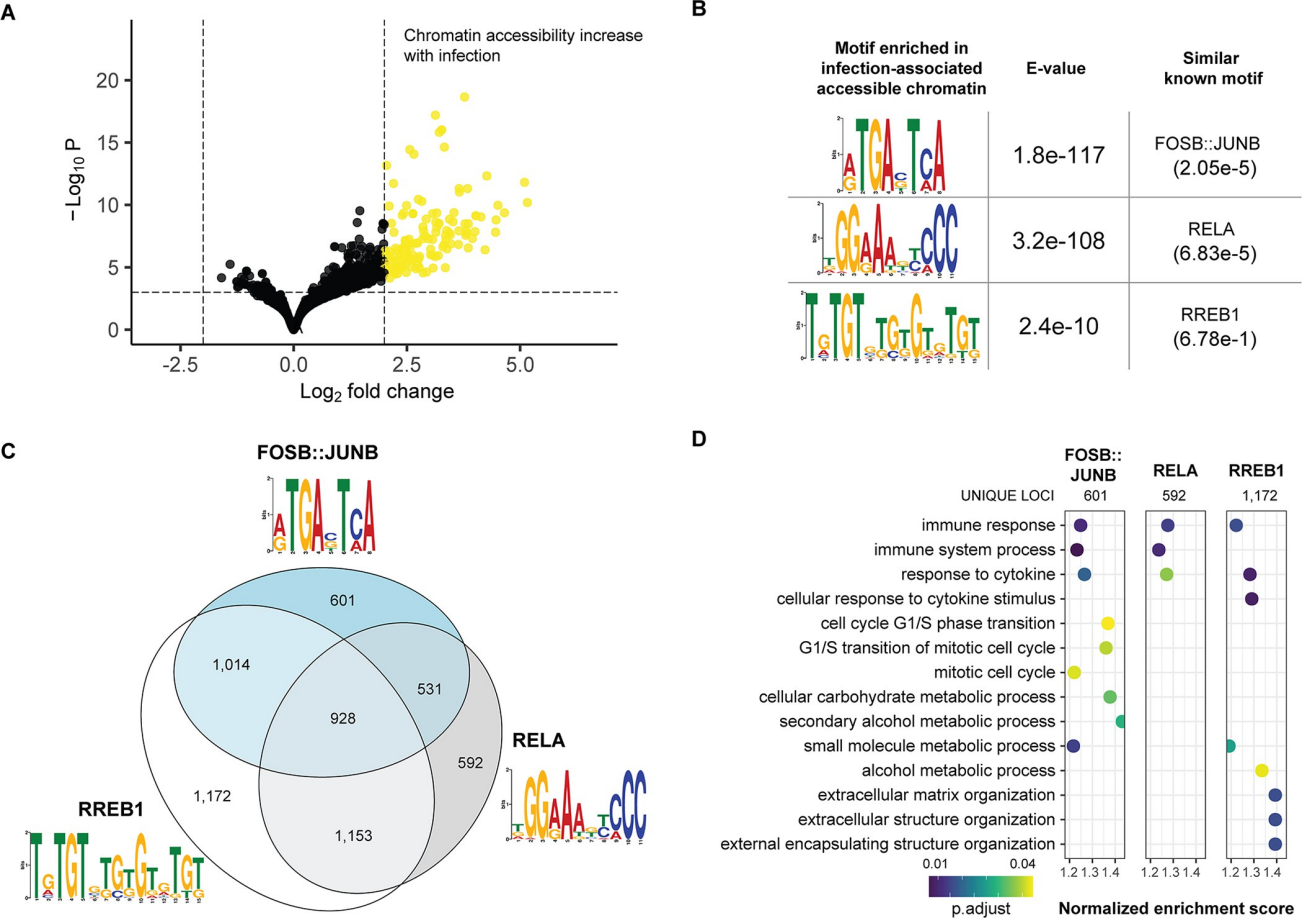
Inferred transcription factors mediating host cell chromatin accessibility changes. In (A) we show the response to *T*. *gondii* infection in the human genome is marked by increased accessibility of chromatin at numerous loci. The results in (B) describe the motifs enriched in these loci that open their chromatin, corresponding to the binding sites of three known TFs. The results in (C) show the numbers of expressed genes containing one or more of the TF binding site motifs within 5 kb of their transcription start site. For those genes with only one motif, testing their ontological properties in (D) revealed the common property of induction of immune responses, but also other properties that are likely to be more favorable to the pathogen.

To gain insights into the mechanism for the host transcriptional changes during *T*. *gondii* infection, we performed the assay for transposase-accessible chromatin (ATAC-seq) [29] in the fibroblasts before and after infection. One infected sample had a pattern of chromatin accessibility more comparable with the uninfected than the other infected samples (**S2 Fig**), in addition to an average of 28% fewer *T*. *gondii* reads, and therefore was removed from the comparison with the interpretation that this sample did not achieve a degree of infection comparable to the other replicates. We used DESeq2 [30] to identify the differentially accessible regions (DARs). Loci gaining chromatin accessibility with infection outnumbered those losing accessibility (503 compared with 27, **Fig 2A**). Of these DARs, only 51 (9.6%) were located within 5 kb of annotated transcription start sites, indicated that chromatin accessibility changes mostly occur at distal regulatory elements (**S3 Fig**).

To gain insights into the transcription factors (TFs) potentially mediating these changes in chromatin accessibility, we performed motif enrichment analysis using MEME-ChIP [31] on the collection of 100 bp sequences flanking the center of the DARs. The motifs discovered were then compared with known TF binding motifs, revealing enrichment at loci opening chromatin during infection for the AP-1 (JUN-FOS), RELA (an NFκB family member) and RREB1 TFs (**Fig 2B**). When we performed an analysis of the same DNA sequences using the vertebrate core motif database JASPAR2020, the same motifs were significantly enriched, accompanied by more NFκB family members and a broader group of candidate TFs (**S4 Fig and S3 Table**).

We asked the question whether these three TFs were distinctively associated with the major groups of differentially expressed genes shown in **Fig 1E–1G**. To maximize our confidence of an association between a DAR and a gene, we focused on those within 5 kb of an annotated gene transcription start site (TSS), identifying DARs containing only one of these motifs (**Fig 2C**), and asked whether binding site motifs for each TF was enriched for genes mediating one or more of the cellular properties altered during infection. We show these results in **Fig 2D**. While all three TF binding motifs were associated with genes mediating immune responses, the RELA (NFκB) motif was enriched at those genes alone, while AP-1 (FOSB::JUNB) motifs were also enriched at genes involved with cell division and metabolic processes, and the motifs for RREB1, which mediates RAS-MAPK signaling [32], are associated with the genes mediating metabolic and extracellular matrix functions. The data could be interpreted to reveal solely host benefits to the NFκB response, but changes that partially benefit the parasite mediated by AP-1 and RREB1.

### The tachyzoite transcriptome of *Toxoplasma gondii* during human host cell infection

**Fig 3:**
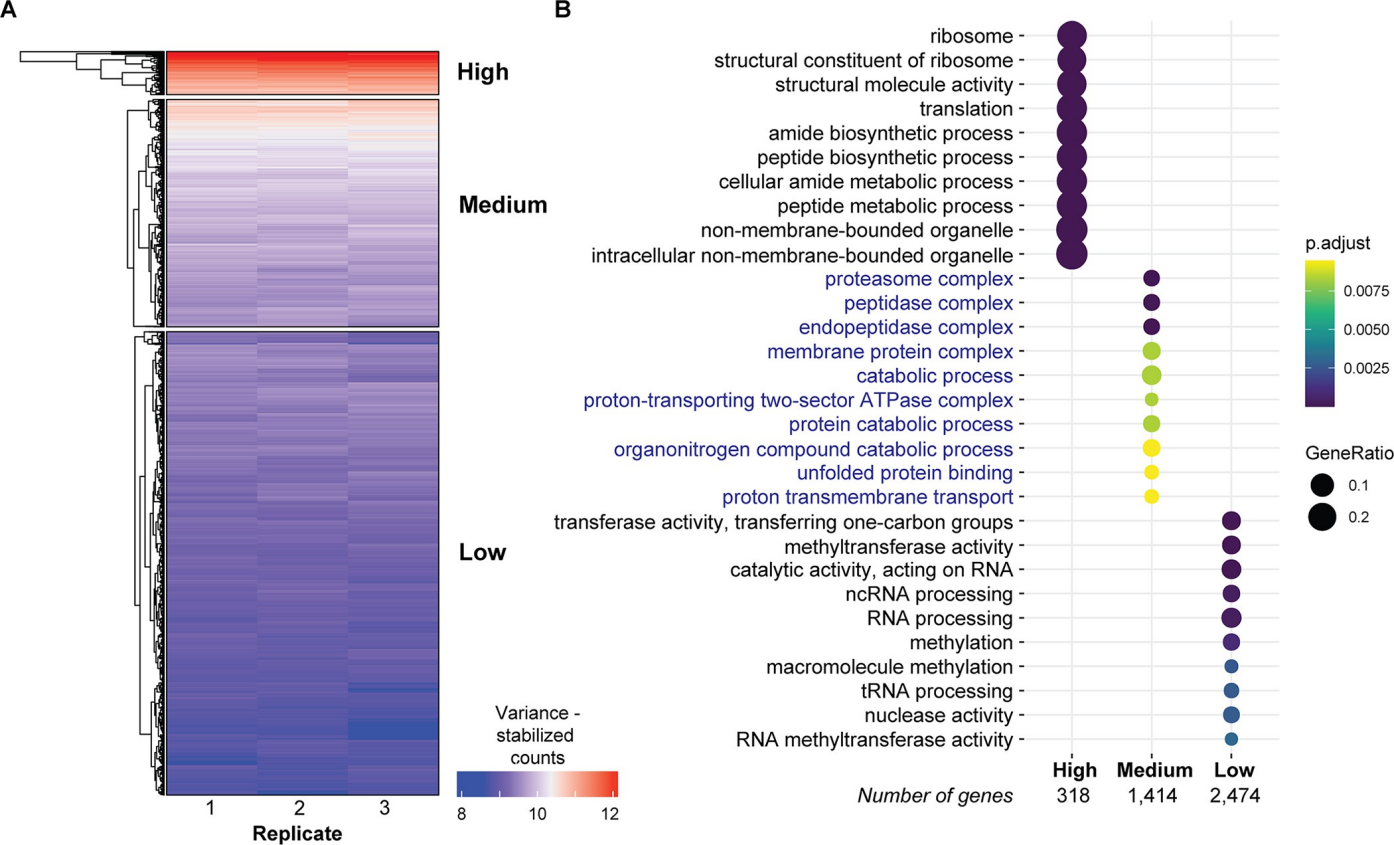
Distinct properties of *T*. *gondii* genes expressed during tachyzoite stage. The *T*. *gondii* transcriptome in its tachyzoite stage while infecting human fibroblasts can be classified into three groups of genes based on their expression levels (A). In (B) we show the ontological properties of the genes in each of these three categories.

Our RNA-seq dataset in infected cells included transcripts from the *T*. *gondii* genome. We could therefore use the ME49 reference genome for *T*. *gondii* to align RNA-seq reads, detecting 3 or more reads in ≥2 replicates at 6,653 genes (75% of all ME49-annotated genes), of which 6,191 genes (93%) showed expression in all three replicates (**S4 Table**). Focusing on this last group of consistently expressed genes, we used hierarchical clustering to identify groups with high (391 genes), medium (4,201 genes), or low (2,061 genes) levels of expression (**Fig 3A**). Using the gene ontology (GO) annotations of *T*. *gondii* ME49 genes from ToxoDB (toxodb.org), we found that highly expressed genes are enriched for protein translation processes, medium expression genes for proteosome and catabolic protein processes, and low expression genes for RNA processing and genomic activity (**Fig 3B, S5 Table**). Overall, our enrichment analysis reveals a hierarchy of biological functions of differentially-expressed genes during the tachyzoite stage of *T*. *gondii* related to their roles during infection.

### *Cis-*regulatory loci in the tachyzoite *Toxoplasma gondii* genome

Using the ATAC-seq dataset from the infected cells, we were able profile the loci with accessible chromatin genome-wide in the *T*. *gondii* genome, using the *T*. *gondii* ME49 reference genome for alignment, representing a follow up to our prior microarray ChIP study of 650 kb of the *T*. *gondii* genome [6], and representing one of only a few genome-wide studies to date of transcriptional regulation in *T*. *gondii* [33–37].

Within the ATAC-seq sample, a mean of 34.6% of reads across the replicates mapped to the *T*. *gondii* ME49 genome. The reads exhibited the expected fragment size distribution characteristic of nucleosome free and nucleosomally-organized fragments (**S5A Fig**), with the exception of reads originating from the apicoplast chromosome, where there is no evidence of nucleosomal organization (**S5B Fig**).

**Fig 4:**
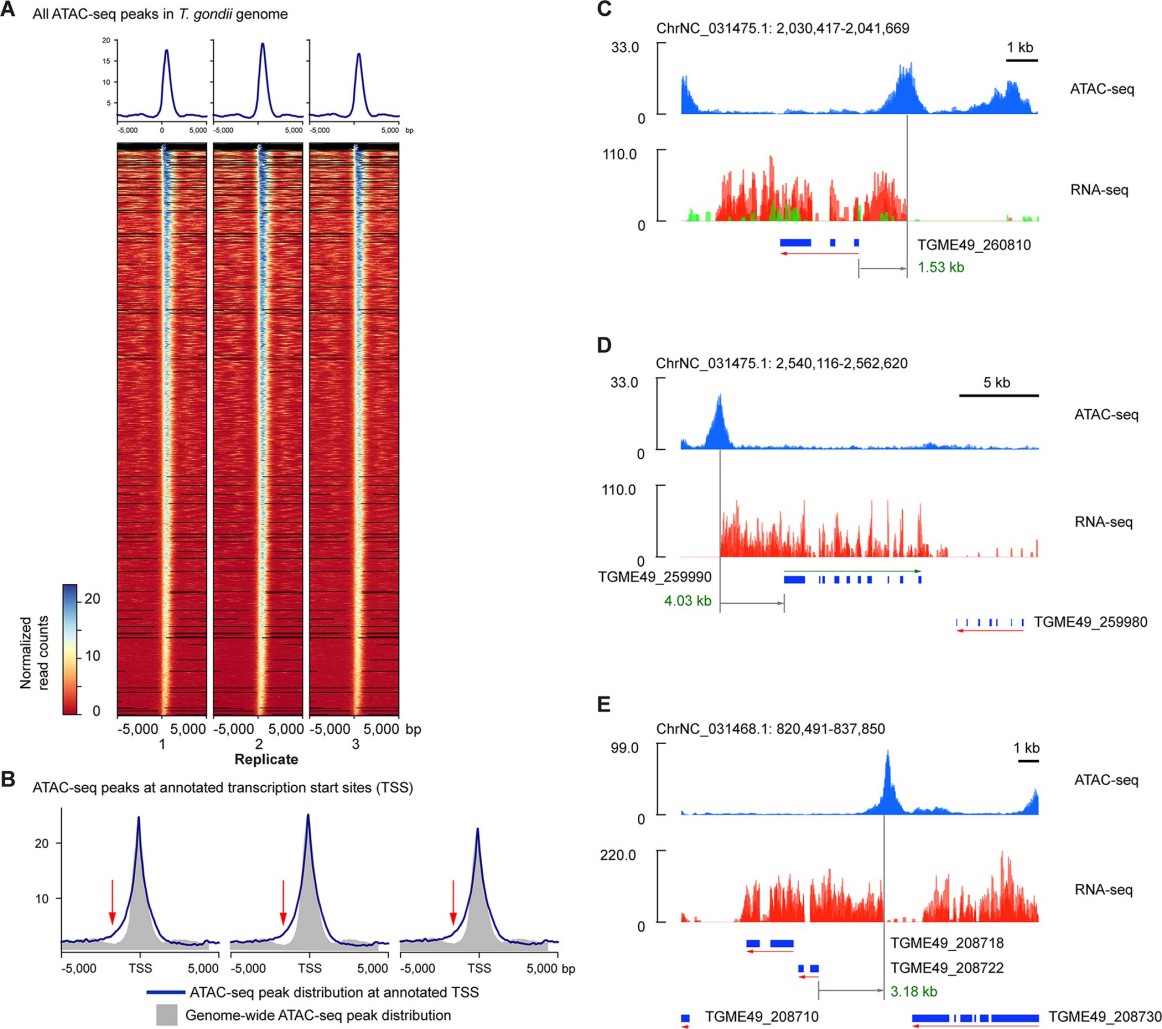
Chromatin accessibility profiles in the *T*. *gondii* genome indicate incomplete annotations of genes. The chromatin accessibility pattern at all of the ATAC-seq peaks in the *T*. *gondii* genome at the tachyzoite stage of its life cycle is shown in (A). When the subset within ±5 kb of annotated *T*. *gondii* transcription start sites (TSS) is plotted with the associated genes represented to the right of the TSS (B, blue line), the patterns shows a skewing relative to the distributions of all peaks from panel (A) (gray distribution). Examples of genes are show to represent how the annotated TSS can be several kilobases (kb) from the upstream ATAC-seq peak and the start of the RNA-seq reads, with examples of 1.53 kb (C) and 4.03 kb (D) differences illustrated. In (E) we gain the additional insight that the two annotated genes are only associated with a single upstream ATAC-seq peak, indicating that there is only one transcript at this locus and the genes could be combined into a single annotation.

A total of 4,672 unique peaks were called across all replicates, with the fraction of reads located within peaks (FrIP score [38]) averaging 61.9%, and an average of 94.9% for peak concordance between replicates. Applying ChIP-R [39], we defined 1,631 peaks as reproducible and focused on these for subsequent analyses (**Fig 4A**). The majority of these peaks (96.1%) were located within 5 kb of an annotated genomic feature, with 91.1% located <1 kb from a transcription start site. When we compared the distribution of ATAC-seq peaks to annotated transcription start sites (TSS), we found a skewing upstream (**Fig 4B**), reflecting many gene annotations for which the TSS is located substantially downstream from the open chromatin region where the start of the RNA-seq reads is also apparent (examples in **Fig 4C**).

**Fig 5:**
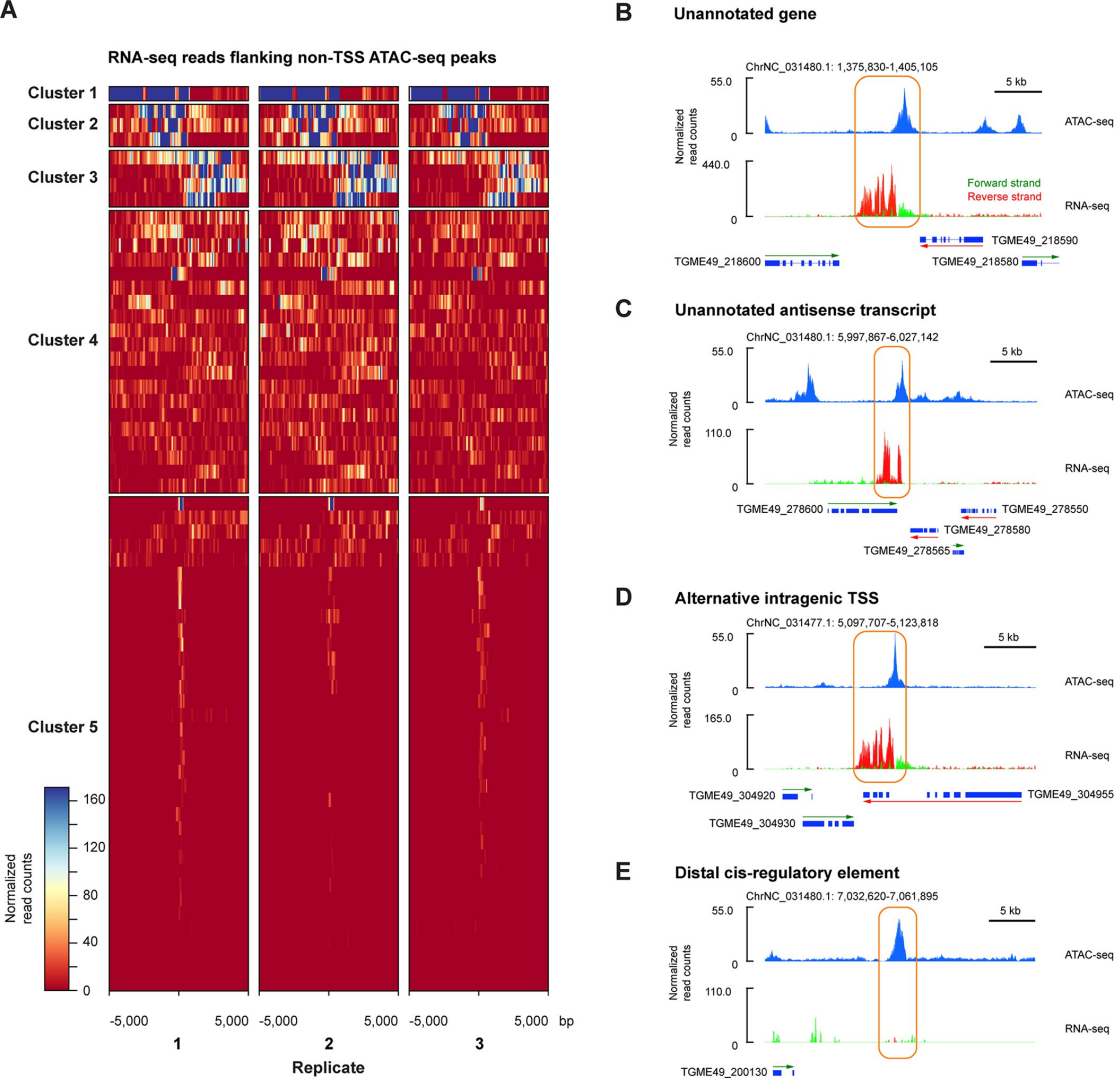
Chromatin accessibility and transcriptional profiling identifies new features in the *T*. *gondii* genome. The gene expression and chromatin accessibility information combine to allow new *T*. *gondii* genomic annotations. In (A) we show the RNA expression at ATAC-seq peaks that are not within 5 kb of an annotated TSS. We sorted these peaks by clustering the characteristics of the RNA-seq data nearby, with the top clusters (1–3 especially) showing evidence for adjacent transcripts. Examples of the loci are shown on the right, including what appears to be a new gene (B), an antisense transcript from the 3’ end of an annotated gene (C), the use of an alternative intragenic promoter (D) and a locus of open chromatin with no nearby genes or RNA expression, potentially representing a distal *cis*-regulatory element.

Some ATAC-seq peaks were not located close to annotated TSS. We explored the representation of RNA-seq reads near this subset of peaks and performed K-nearest neighbors (KNN) clustering to define 5 clusters (**Fig 5A**). Inspecting these loci, we found examples of what appear to be new, unannotated genes (**Fig 5B**), unannotated anti-sense transcripts (**Fig 5C**), the use of alternative TSS within bodies of annotated genes (as previously noted by Markus and colleagues [35], **Fig 5D**) and distal *cis*-regulatory elements (**Fig 5E**). The combination of ATAC-seq and RNA-seq appears to permit more detailed annotation of the genome than would each assay on its own.

### Candidate *Toxoplasma gondii* transcription factor binding motifs

**Fig 6:**
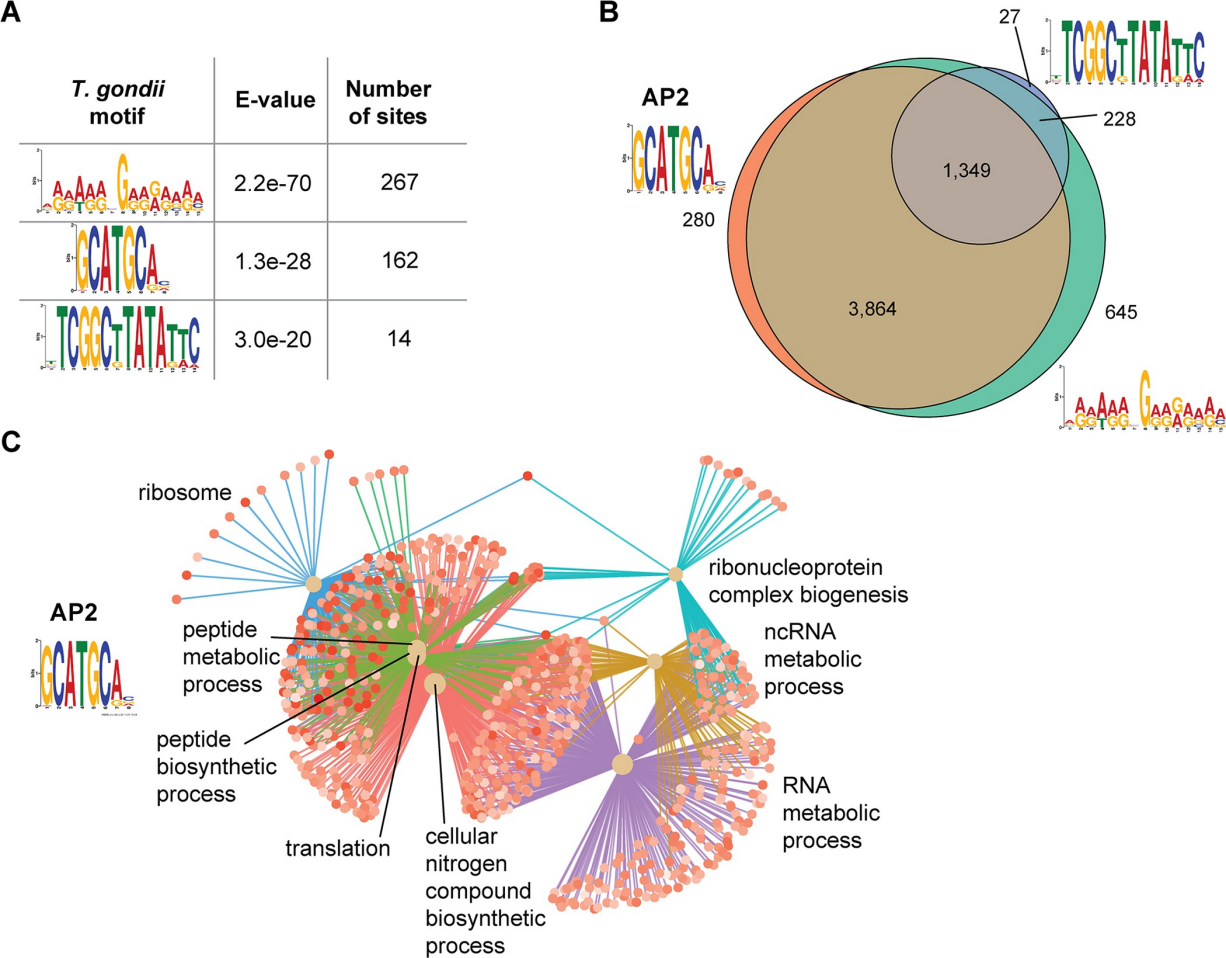
Inference of transcription factor binding motifs in the *T*. *gondii* genome. Analysis of the loci of open chromatin in the *T*. *gondii* genome reveals enrichment for several motifs (A). The purine (AG)-rich motif is the most abundantly represented at *T*. *gondii* promoters (B) followed by the known AP2 motif (GCATGCA), with the TATA-containing motif in 25.1% of promoters. Panel (C) shows the properties of the subset of genes with the AP2 motif at their promoters.

The chromatin accessibility dataset provided a novel opportunity to define candidate TF binding sites involved in *T*. *gondii* transcriptional regulation. We used *de novo* motif enrichment analysis with MEME-ChIP [31] and identified three significant (e-value <1e^-20^) motifs (**Fig 6A**).

The most abundant *T*. *gondii* motif that we identified is the purine-rich AAAAAGGAAGAAA sequence, located at 267 open chromatin regions. The next most abundant motif that we found is the known apicomplexan AP2 TF binding site GCATGCA, first found in other apicomplexan parasites, *Plasmodium falciparum*, *Theileria*, and *Cryptosporidium* [40–42]. The third motif contains a strong TATA sequence, potentially representing the TATA box that has proven elusive in prior *T*. *gondii* studies [43].

We found the three motifs to be frequently co-located within the same gene promoters. In 1,349 genes all three of the motifs were found in the promoter region (**Fig 6B**). The candidate TATA box-containing motif is present at the promoters of 25.1% of *T*. *gondii* genes, comparable to the estimates of 20% of yeast promoters [44] and 27% of human promoters [45] having TATA boxes. When we analyzed the genes containing the better characterized AP2 motif, we found them to be significantly enriched for RNA and protein metabolic and biosynthetic processes (**Fig 6C**).

### Potential *Toxoplasma gondii* transcription factor effects on host cell chromatin

Finally, we asked the exploratory question whether there was any evidence that TFs from *T*. *gondii* could be accessing the chromatin of the host cell and directly altering transcription, extending prior models of *T*. *gondii* effects on host chromatin [46–48]. We used the three major *T*. *gondii* TF motifs of **Fig 6** and explored whether any of these were enriched at loci where chromatin becomes accessible during infection. We found 46 loci in the host genome to meet these criteria **(S6 Table)**. In **S7 Fig** we show three examples, the first two (panels A-B) showing the AP2 motif at loci that open chromatin at intronic regions of the *LINC02703* and *PCMTD1* genes, while panel C shows the purine-rich motif at the bidirectional promoter of *TENM3-AS1/ ENSG00000248266*, with chromatin opening at this locus associated with increased local transcription. While these findings could certainly represent a chance occurrence of overlap of the *T*. *gondii* TF motifs at loci that are undergoing chromatin remodelling during infection purely due to host TF activities, we speculate that it is possible that *T*. *gondii* TFs access the host nucleus and influence host chromatin states directly. The data presented define candidate loci for further exploration.

## Discussion

This is the first study that looks simultaneously at the transcriptome and chromatin organization of both the host cell and an intracellular apicomplexan parasite during infection. Our goal was to gain insights into the interaction between the activity of the well-studied parasite *T*. *gondii* and the response of the infected human host cell, by studying gene expression and chromatin responses concurrently. We note that there have been elegant prior transcriptional [49, 50] and chromatin studies [33, 51, 52], and promoter mapping [35, 53] of apicomplexan organisms, and of host cell transcriptional responses [54] that show differences between cell types [55], a reminder that our results should be considered specific to infection of human fibroblasts. Our intent was to extend these studies to gain insights into the conflict being fought at the molecular level during infection by this very successful infectious agent.

The host gene expression changes are abundant, and mirror the results of several prior studies, including the induction of immune response genes [56], and the over-expression of matrix metalloproteases [57], while the induction of metabolic reprogramming is consistent with the auxotrophic nature of *T*. *gondii*, and the mitotic gene response reflective of prior observations of enhancement of cell cycle progression following *T*. *gondii* infection [58]. In order to understand how these responses are regulated, we were able to use TF binding motif information in the open chromatin regions of the host cell to infer the TF responses mediating these transcriptional changes. This analysis provided the insight that the NFκB response appeared to be predominantly involved in host cell immune responses, but JUN:FOS (AP-1) and RREB1 are likely to have additional properties of cell division, metabolic processes, and extracellular matrix gene regulation.

**Fig 7:**
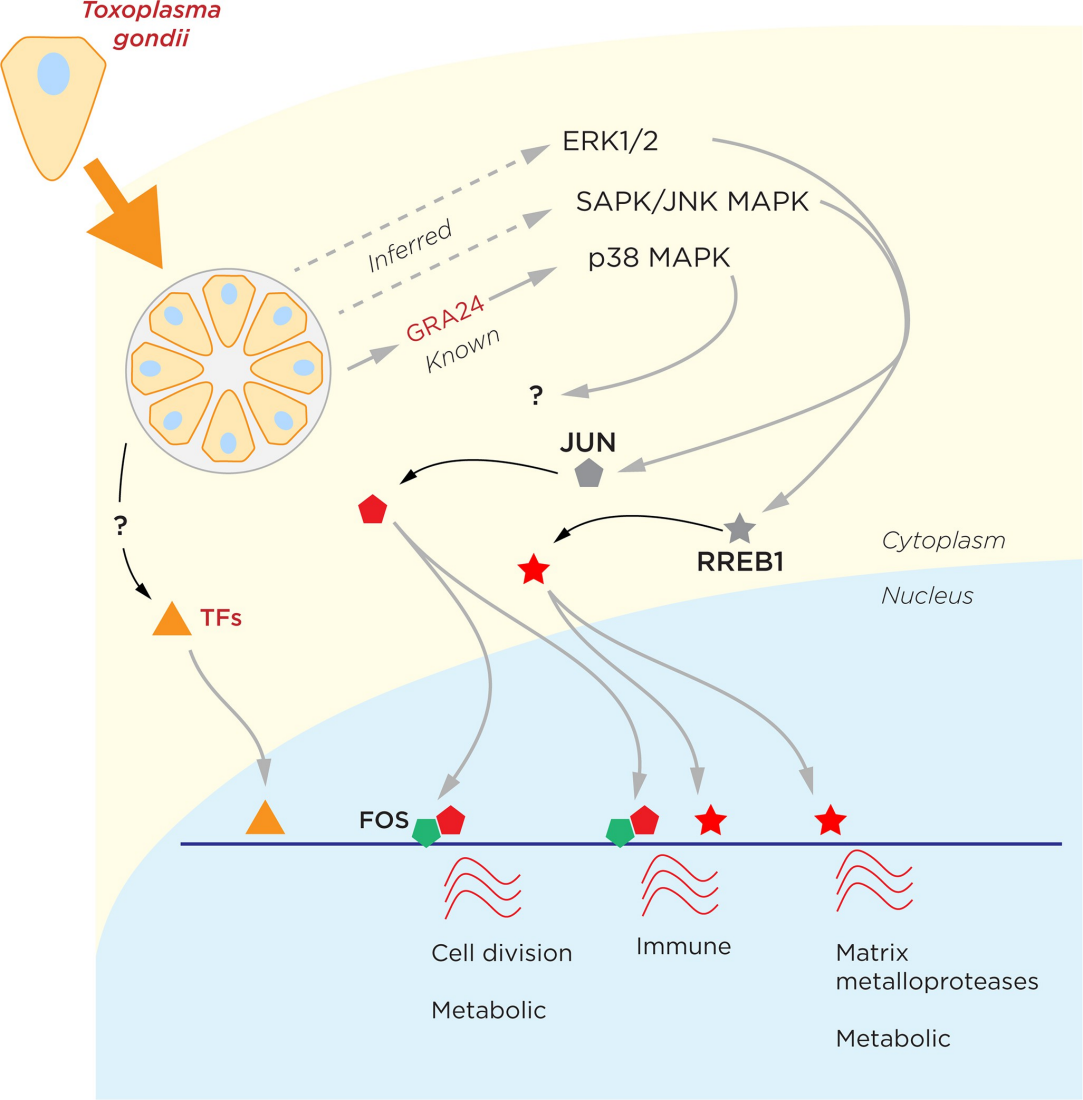
A model derived from genomic assay data for the host cellular response to *T*. *gondii* infection. By inferring the TFs mediating the host cell response, we can further predict the cell signaling pathways induced by *T*. *gondii* infection in human fibroblasts. How the known GRA24 induction of p38 MAPK signaling influences the transcriptional response remains uncertain, and we include the possibility that *T*. *gondii* TFs may contribute to host cell transcriptional dysregulation.

We were then able to add further step of inference by asking how these TFs could be altered in their functions with *T*. *gondii* infection. It is known that *T*. *gondii* exports into the host cell the GRA24 protein, which complexes with [59] and induces mitogen-activated protein (MAP) kinase p38α (P38 MAPK) [60]. The TFs that respond to P38 MAPK signaling include ATF1/ATF2, CEBP family members, SAP1, p53 and ETS1 [61], but their associated binding motifs were not prominent at loci gaining chromatin accessibility, making it unclear how GRA24 exerts transcriptional effects during infection. In **Fig 7** we present a model for cell signaling pathways influencing JUN and RREB1, implicating the ERK1/2 and SAPK/JUNK MAPK signaling that are known to be induced by *T*. *gondii* infection [62, 63] in the induction of these TFs. These signaling pathways are also activated in *Leishmania* [64], *Plasmodium* and *Theileria* [65] infections, indicating how central these are in the host response to protozoal infections. The downstream response to the activation of these JUN and RREB1 TFs involves host responses likely to be favorable to the pathogen, and we also depict how there may also be the possibility that *T*. *gondii* TFs directly influence host cell chromatin (**S6 Fig**). This **Fig 7** model is undoubtedly incomplete but serves to illustrate how these genomic assay data can be used to infer a lot more about the pathogen-host interaction than just host transcriptional outcomes alone.

The data also reveal new insights into the *T*. *gondii* genome. We observe that stratification of tachyzoite gene expression by expression levels correlates with function. Mapping ATAC-seq peaks generally enriches the region close to the annotated transcription start site (TSS) of *T*. *gondii* genes, but we see numerous examples of open chromatin located at substantial distances from TSSs (**Fig 4**), providing a resource for improved TSS/promoter annotation in the *T*. *gondii* genome. We also find that there are ATAC-seq peaks more than 5 kb from any TSS, and show in **Fig 5** how the combination of ATAC-seq and RNA-seq data allows new genes, antisense transcripts, intragenic alternative promoters and distal *cis*-regulatory elements to be predicted, a resource for enhancing the annotation of the *T*. *gondii* genome.

Motif analysis of the loci of open chromatin in *T*. *gondii* reassuringly rediscovered the known AP2 TF motif [40–42]. We also found enrichment of a purine-rich motif of uncertain significance present at a large proportion of TSS, a motif not previously identified in the few studies of *cis*-regulatory loci in *T*. *gondii*. The presence of the TATA motif within the TCGGCTTATATTC sequence raises the possibility that *T*. *gondii* does, in fact, use a TATA box [43].

In summary, the combined study of host and parasite transcription and its regulation offers insights into the conflict between the host and pathogen, with some evidence for host physiology being harnessed by the pathogen for its own purposes. The ability to infer through molecular genomic studies insights into cell signaling is relatively novel and typically an underutilized potential insight from molecular genomics studies, and is made possible by focusing on TFs as the mediators of molecular genomic changes.

## Materials and methods

### Cell and parasite cultures

Cell and parasite cultures were maintained as previously described [66]. Human Foreskin Fibroblasts (HFFs) were grown in Dulbecco’s modified Eagle medium (DMEM) supplemented with 10% fetal bovine serum, 100 U/mL penicillin, 100 μg/mL streptomycin and 2 mM L-glutamine and were maintained at 37°C with 5% CO_2_. *T*. *gondii* Type I tachyzoites (RHΔHxΔKu80) were also maintained at 37°C with 5% CO_2_ by infecting 25 cm^2^ flasks containing confluent HFFs every 48 hours.

### Host cell infections

The 25 cm^2^ flasks containing freshly egressed *T*. *gondii* tachyzoites were then passaged through a 25-gauge needle three times to lyse out any remaining intracellular tachyzoites from their hosts, and the tachyzoites were spun down at 3,000 rpm for 8 minutes. 75 cm^2^ flasks containing confluent HFFs were then infected with these tachyzoites using a multiplicity of infection (MOI) of 3, and 24 hours after the infection the percentage of infected cells per flask was calculated. Flasks counted as at least 80% infected at this time were harvested by scraping. A flask containing HFFs to be used as an uninfected control was also harvested in parallel by scraping. The cells were pelleted by centrifugation at 1,300 rpm for 5 minutes. The cells were harvested such that both RNA and DNA could be extracted from the same flask for each biological replicate, and a total of three biological replicates of both the control and infected flasks were harvested in total for each assay.

### DNA extraction

Genomic DNA (gDNA) was extracted from each sample as follows: 500 μL 10% SDS and 10 μL RNase A (10 mg/mL) were added to each sample followed by an incubation at 37°C for 1 hour. 50 μL of proteinase K (20 mg/mL) was then added and the samples were incubated at 55°C overnight. An equal volume of saturated phenol was then added to each tube, and the samples were mixed slowly at room temperature for 15 minutes. The samples were then spun down at 3,000 rpm for 10 minutes and the supernatant was collected. A total of 3 phenol extractions were performed, followed by 2 chloroform extractions. The samples were then pipetted into a dialysis bag and further purified at 4°C through three changes of a 20x NaCl, sodium citrate (SSC) buffer over the course of 24 hours. Dehydration was then performed by covering each sample in a pile of polyethylene glycol (PEG) for ~1 hour. The PEG was rinsed from each dialysis bag and the gDNA extracted from each sample was collected.

### RNA extraction

RNA was extracted using TRIzol reagent (Invitrogen Cat. # 15596018) according to the manufacturer’s instructions as follows. One milliliter of TRizol was added to each sample and incubated at room temperature for 5 minutes. A volume of 200 μL of chloroform was added to each sample and the tubes were shaken vigorously for 15 seconds and then incubated for 2–3 minutes at room temperature. The samples were then centrifuged at 12,000 rpm for 15 minutes at 4°C, and the aqueous phase of each sample were collected. Following this, 0.5 mL of 100% isopropanol was added to the aqueous phase, and the samples were incubated for 10 minutes at room temperature, followed by centrifugation at 12,000 g for 10 minutes at 4°C. The supernatant was removed from each tube and the pellet was washed in 1 mL of 75% ethanol and centrifuged at 7,500 g for 5 minutes at 4°C. The supernatant was again removed and the pellet was air dried for 5 minutes. The RNA pellet was then resuspended in RNase-free water and incubated at 55°C for 10 minutes. Following the RNA extraction, the samples were then treated with DNase to remove contaminating gDNA, and further purified using the miRNeasy Mini Kit (QIAGEN cat# 217004). The Bioanalyzer was used to assess total RNA integrity prior to library preparation, and only samples with an RNA integrity number (RIN) greater than 8 was used for further downstream library preparation.

### Directional RNA-seq library preparation

Directional RNA-seq libraries were prepared as previously described by our lab [67]. One microgram of RNA was DNase-treated and rRNA-depleted (Ribozero rRNA Removal Kit, Epicentre). RNA was used as a template for SuperScript III first-strand cDNA synthesis (ThermoFisher Scientific, SuperScript III kit cat# 18080–044), using oligo-dT as well as random hexamers. Actinomycin D was added to the reaction to prevent any possible amplification from contaminating gDNA. During second-strand synthesis, a (dUTP/dATP/dCTP/dGTP) mix was used to create directional libraries. Before library preparation, cDNA samples were Covaris- fragmented to 300 bp fragments. The samples were then end-filled, 3’ terminal A extended and ligated with TruSeq-indexed Illumina adapters. Uracil-DNA-glycosylase (UDG) treatment preceded the PCR reaction to amplify exclusively the originally oriented transcripts. Libraries were amplified using P5 and P7 Illumina primers. Prior to sequencing the libraries were gel- extracted for size selection, primer-dimers were removed and the quality was assessed on the Bioanalyzer. The six libraries were multiplexed on a single lane of the Illumina Hi-Seq 2500 platform twice to obtain 100 bp single-end reads. The reads combined from the two lanes of the sequencer for each sample were used for further downstream analysis, resulting in a mean of 25 million reads per sample **(S7 Table)**.

### ATAC-seq library preparation

The assay for transposase accessible chromatin (ATAC)-seq libraries were prepared as previously described [29]. A total of three biological replicates each of uninfected and *T*. *gondii* infected HFFs were harvested at 24 hours post-infection. To prepare the nuclei, we spun 75,000 cells at 500 g for 5 minutes. Each sample was washed using 50 μL of cold 1x PBS. Samples were then centrifuged at 500 g for 5 minutes. Cells were lysed in cold lysis buffer (10 mM Tris- HCL, pH 7.4, 10 mM NaCl, 3 mM MgCl_2_ and 0.1% IGEPAL CA-630). Immediately following lysis, nuclei were spun at 500 g for 10 minutes at 4°C. The pellet was then resuspended in the transposase reaction mix (25 μL 2x TD buffer, 2.5 μL transposase and 22.5 μL nuclease-free water).

The transposition reaction was carried out for 30 minutes at 37°C and the samples were then purified using a Zymo DNA Clean and Concentrator purification kit. Following the purification, we amplified the libraries with the following PCR conditions: 72°C for 5 minutes; 98°C for 30 seconds; and a total of 10 cycles of 98°C for 10 seconds; 63°C for 30 seconds; 1 minute for 72°C. Libraries were purified using Agencourt AMpure beads. Libraries were further amplified using the PCR conditions: 98°C for 30 seconds; and a total of 14 cycles of 98°C for 10 seconds; 63°C for 30 seconds and 72°C for 1 minute. The six ATAC-seq samples were multiplexed on a single lane of the Illumina Hi-Seq 2500 to obtain 100 bp paired-end reads, which resulted in a mean of 53 million reads per sample **(S8 Table)**.

### RNA-seq read processing, alignment, and quantification

101 bp single-end reads were trimmed to remove low quality base calls and Illumina universal adapters using Trim Galore! (version 0.6.5) with default parameters and then assessed using fastQC (version 0.11.4) and multiqc (version 1.10.1) [68]. Trimmed reads were aligned to a combined human (GRCh38), human decoy (GRCh38), and *T*. *gondii* (ME49) genome using Hisat2 (version 2.0.4) [69] with default parameters. Reads were then split into human and *T*. *gondii* fractions using samtools (version 1.9) [70]. Aligned reads were then assessed for each organism separately using RSeQC (version 2.6.4) [71] and Qualimap (version 2.2.1) [72]. Aligned reads were then quantified using HTseq (version 0.12.3) [73] in union mode using default parameters. Alignment tracks were generated for forward and reverse strand reads using deepTools (version 3.1.0) [74] normalized using scaling factors calculated using the RLE method in DESeq2 (version 1.32) [30], and viewed in IGV (version 2.9.4) [75].

### Human and *Toxoplasma gondii* differential expression analysis

Differential expression analysis was performed using DESeq2 and gene set enrichment analysis performed using clusterProfiler (version 4.0.5) [76]. A gene ontology annotation for *T*. *gondii* ME49 was downloaded from ToxoDB. For *T*. *gondii*, only the infected replicates were analyzed.

### ATAC-seq read processing, alignment, and quantification

101 bp paired-end reads were trimmed to remove low quality base calls and Illumina universal adapters using Trim Galore! (version 0.6.5) with default parameters and then assessed using fastQC (version 0.11.4) and multiqc (version 1.10.1). Trimmed reads were aligned to a combined human (GRCh38), human decoy (GRCh38), and *T*. *gondii* (ME49) genome using bwa mem (version 0.7.17) [77] with default parameters. Samtools (version 1.9) [70], Picard tools (version 1.92), and deeptools (version 3.1.0) [74] were used for removing mitochondrial reads, PCR optical duplicates, low quality reads, and shifting reads to account for Tn5 transposase offset. ATACSeqQC (version 1.16) [78] was used to assess aligned reads. Narrow peaks were called using MACS2 (version 2.1.0) [79] using default parameters for each replicate and reproducible peaks across all replicates in the same condition were called using ChIP-R (version 1.1.0) [39]. Reproducible peaks from each condition were combined into non-redundant peaks, blacklisted regions were filtered, and peaks were quantified using Rsubread (version 2.6.4) [80] and GenomicAlignments (version 1.28.0) [81]. Alignment tracks were generated using deepTools, normalized using scaling factors calculated using the RLE method in DESeq2, and viewed in IGV.

### Human and *Toxoplasma gondii* differential accessibility analysis

Differential accessibility analysis was performed using DESeq2 and gene set enrichment analysis was performed on annotated peaks using clusterProfiler. A gene ontology annotation for *T*. *gondii* ME49 was downloaded from ToxoDB (toxodb.org). For *T*. *gondii*, only the infected replicates were analyzed.

### Human peak annotation and motif enrichment

Differentially accessible peaks were annotated using ChipPeakAnno (version 3.26.4) [82] with parameters adjusted to output all overlapping features within 5 kb upstream or downstream of the nearest transcription start site. For *de novo* motif discovery, 100 bp fragments were extracted from the middle of the top 1,000 differentially accessible upregulated or downregulated peaks and input into MEME-ChIP (version 5.4.1) [83] with default parameters. chromVAR (version 1.14.0) [84] was used with the JASPAR2020 vertebrate core motif database [85] to identify variable motifs between conditions, testing all differentially accessible peaks.

### *Toxoplasma gondii* peak annotation and motif enrichment

Reproducible peaks in the infected condition were annotated using ChipPeakAnno with parameters adjusted to output all overlapping features within 5 kb upstream or downstream of the nearest transcription start site. For *de novo* motif discovery, 100 bp fragments were extracted from the middle of all reproducible peaks in the infected condition and input into MEME-ChIP with default parameters. To detect potential *T*. *gondii*-specific transcription factors acting on the human genome, *de novo* motifs were searched against the human differentially accessible peak sequences using FIMO (version 5.4.1) [86].

## Supporting information

S1 Fig The gene set enrichment analysis-derived Gene Ontology (GO) terms characterizing the properties of the genes increasing expression with *T*. *gondii* infection. (TIF)

S2 Fig A PCA plot of ATAC-seq of human host cell genes, using the 5,000 most variable peaks across all replicates. The analysis shows that one infected sample (I1) clusters with the uninfected samples (U1-3). We interpreted this to indicate that the infection in this replicate was poor, and excluded this I1 sample from further analyses.
(TIF)

S3 Fig The location of ATAC-seq peaks (left) and differentially-accessible regions (DARs, right) relative to annotated transcription start sites (TSS) in the human genome. Whereas ATAC-seq peaks are strongly enriched at TSS, only 51 (9.6%) of DARs are located within 5 kb of annotated transcription start sites (yellow shading).
(TIF)

S4 Fig A heat map illustrating the enrichment of transcription factor binding site motifs from a JASPAR analysis of the loci where chromatin becomes more accessible with infection. We see the RELA, RREB1 and FOSB::JUNB motifs now accompanied by many other transcription factor motifs, including many NFκB family members and AP-1 targets. These are all listed in **S3 Table**.
(TIF)

S5 Fig In (A) we show the number of RNA-seq reads from *T. gondii* to be consistently greater than 2 million in all replicates. In (B) we illustrate how most genes in the *T. gondii* genome are represented consistently across replicates, also showing this quantitatively in the UpSet plot of panel (C).
(TIF)

S6 Fig The expected nucleosomal periodicity pattern of chromatin is revealed from the insert size plots of panel (A), representing the reads aligning to the *T. gondii* nuclear genome, whereas in (B) we see no evidence for such nucleosomal organization in reads mapping to the *T. gondii* apicoplast DNA.
(TIF)

S7 Fig We show examples of loci where the motifs enriched at *T*. *gondii* loci of open chromatin are also found in the host genome at sites that open chromatin during *T*. *gondii* infection, representing candidate loci where *T*. *gondii* TFs may be acting directly on host chromatin. (TIF)

S1 Table The list of host genes changing expression with *T*. *gondii* infection. (XLSX)

S2 Table Gene set enrichment analysis-derived Gene Ontology (GO) terms characterizing genes increasing expression with *T*. *gondii* infection. (XLSX)

S3 Table The results of our analysis of loci of open chromatin in infected cells using the vertebrate core motif database JASPAR2020, the data used for S4 Fig. (XLSX)

S4 Table The detailed expression data for the ME49 reference genome annotated genes. We present a raw counts file with data for each replicate, and the normalized counts file on which our analyses were based.
(XLSX)

S5 Table Gene ontology (GO) annotations of *T*. *gondii* ME49 genes from ToxoDB (toxodb.org) applied to genes in expression level categories of high, medium and low. (XLSX)

S6 Table The candidate transcription factor binding site motifs from Fig 6 were used to identify loci in the host human genome where these motifs are present and chromatin becomes accessible during infection, defining candidate loci where *T*. *gondii* transcription factors may be directly acting on the host human genome. We list the 46 candidate loci and the associated motifs.
(XLSX)

S7 Table RNA-seq sequencing data metrics for the human and *T*. *gondii* genomes. (XLSX)

S8 Table ATAC-seq sequencing data metrics for the human and *T*. *gondii* genomes. (XLSX)
