# Supplementary figures and images for: Genomic insights into host and parasite interactions during intracellular infection by Toxoplasma gondii
Source: PLoS One. 2022 Sep 30;17(9):e0275226. doi: 10.1371/journal.pone.0275226 (PMC9524707; doi:10.1371/journal.pone.0275226)

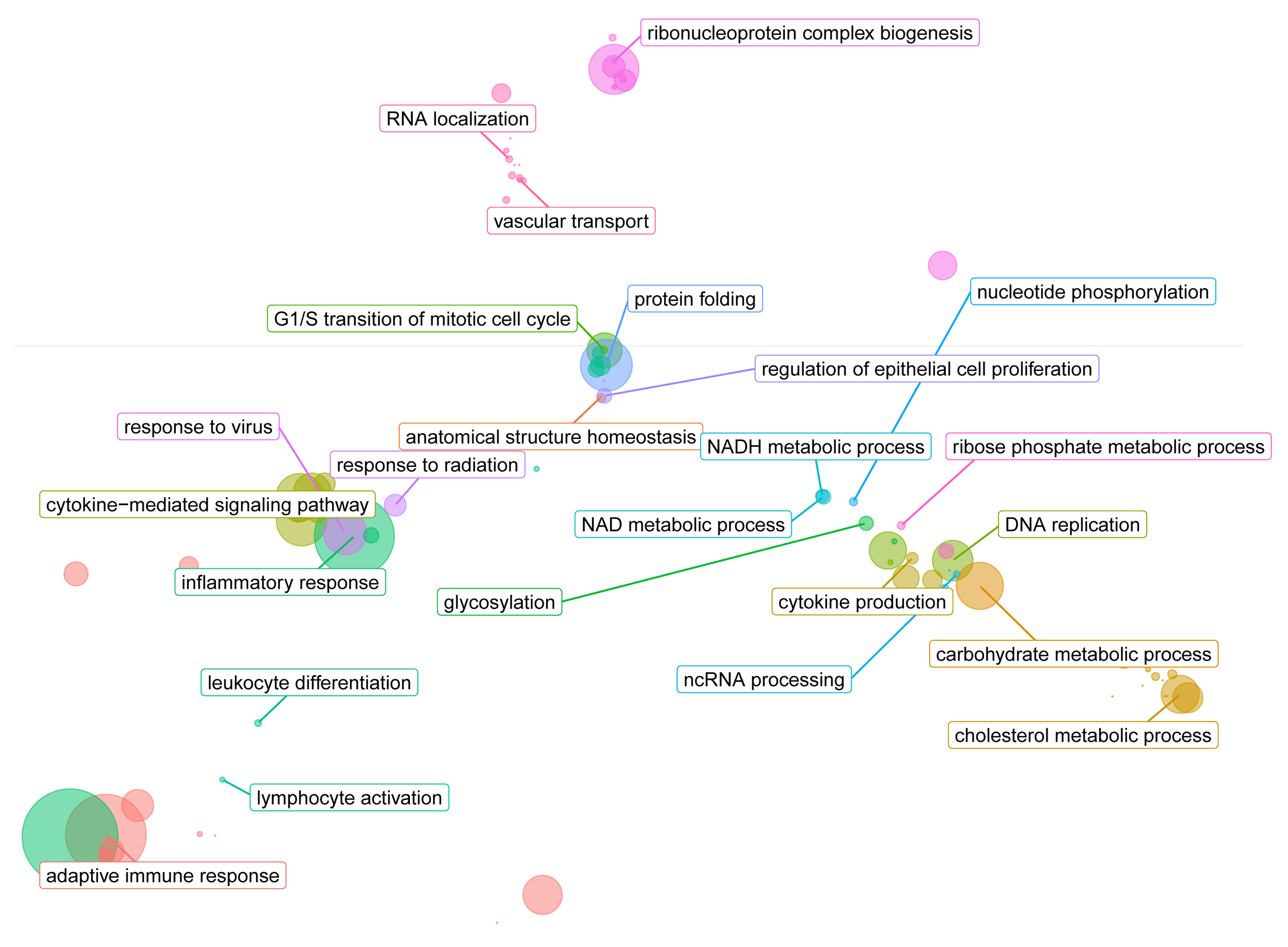

Supplement: S1 Fig — (TIF) [file pone.0275226.s001.tif]

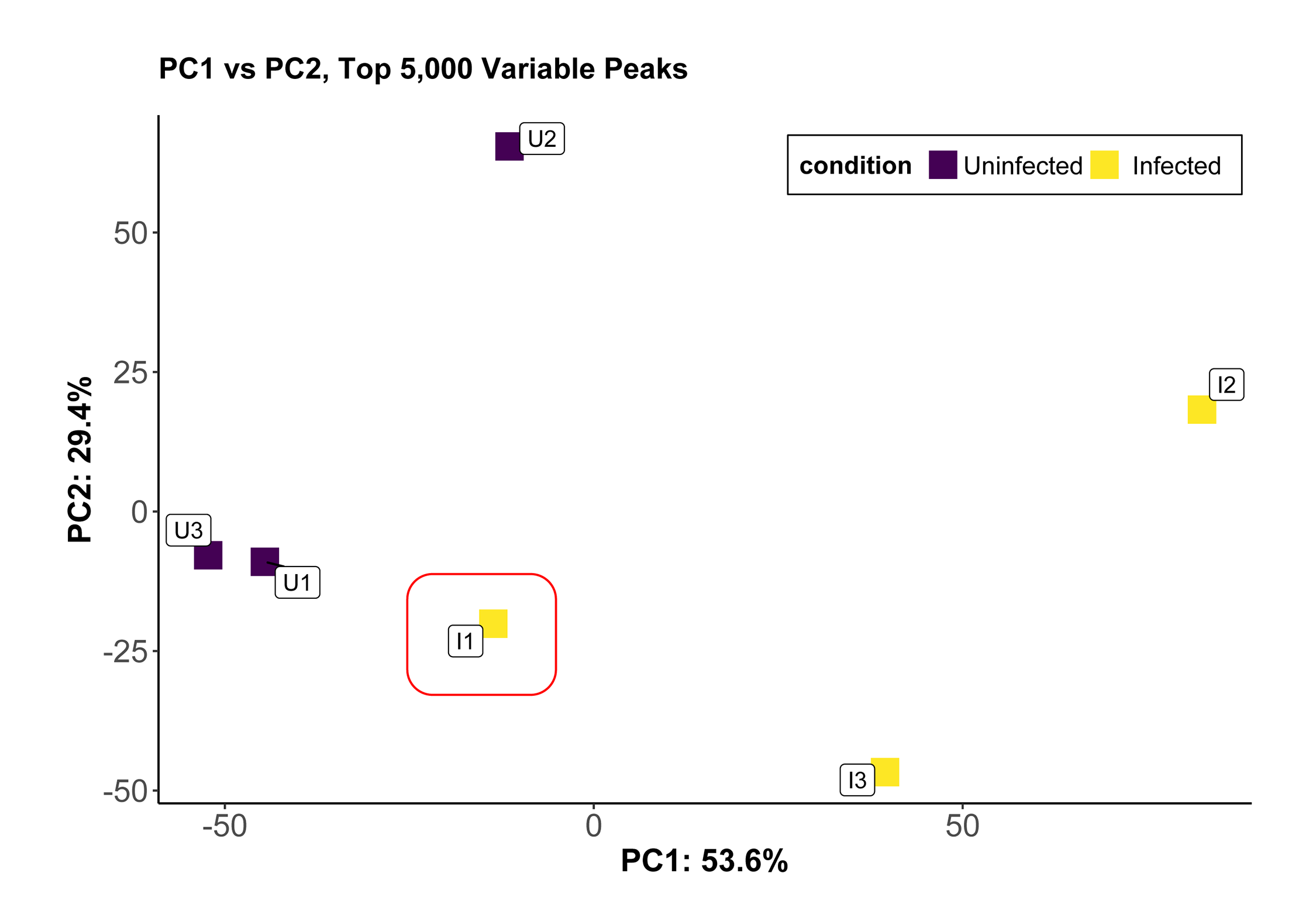

Supplement: S2 Fig — The analysis shows that one infected sample (I1) clusters with the uninfected samples (U1-3). We interpreted this to indicate that the infection in this replicate was poor, and excluded this I1 sample from further analyses. (TIF) [file pone.0275226.s002.tif]

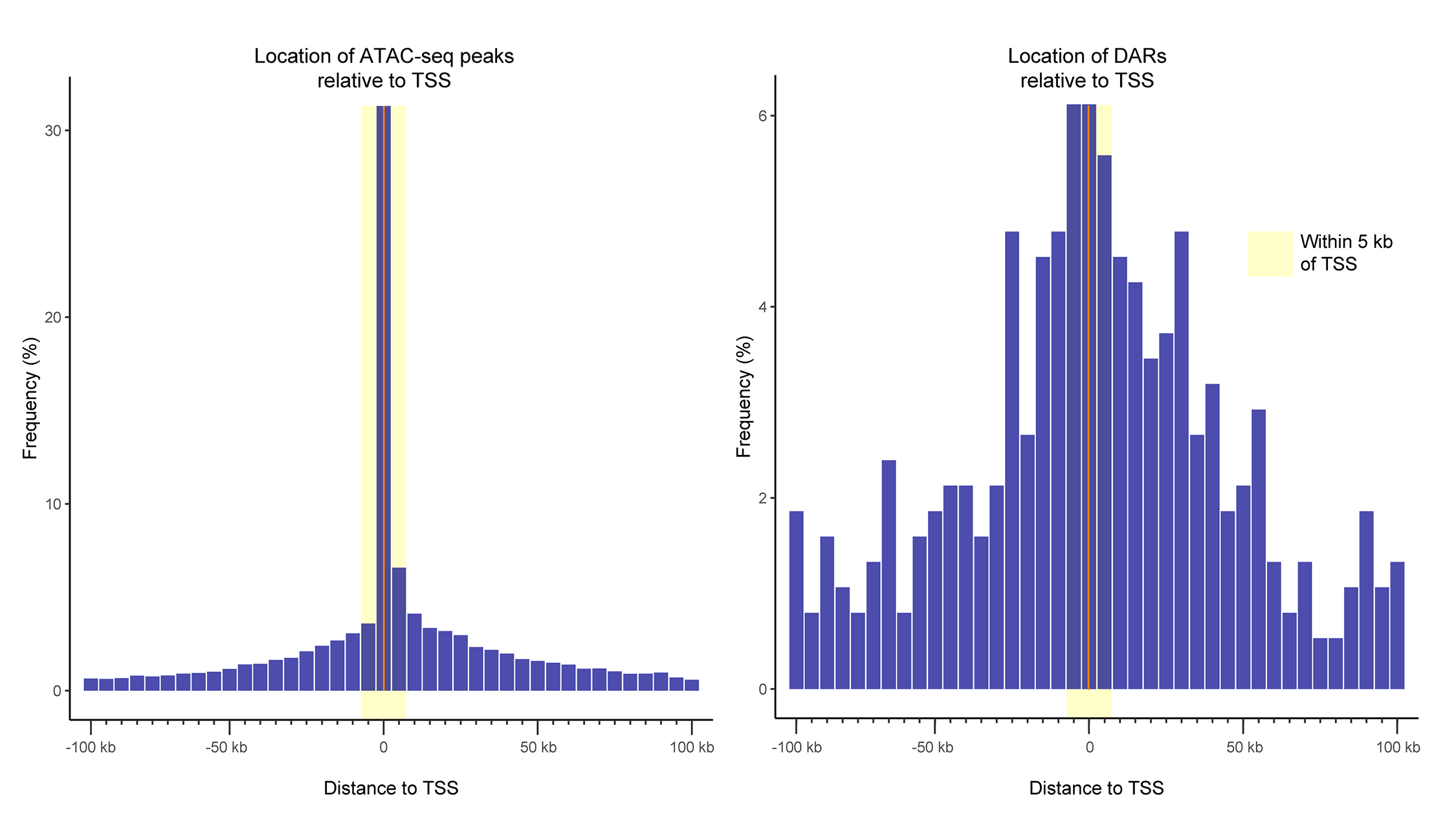

Supplement: S3 Fig — The location of ATAC-seq peaks (left) and differentially-accessible regions (DARs, right) relative to annotated transcription start sites (TSS) in the human genome. Whereas ATAC-seq peaks are strongly enriched at TSS, only 51 (9.6%) of DARs are located within 5 kb of annotated transcription start sites (yellow shading). (TIF) [file pone.0275226.s003.tif]

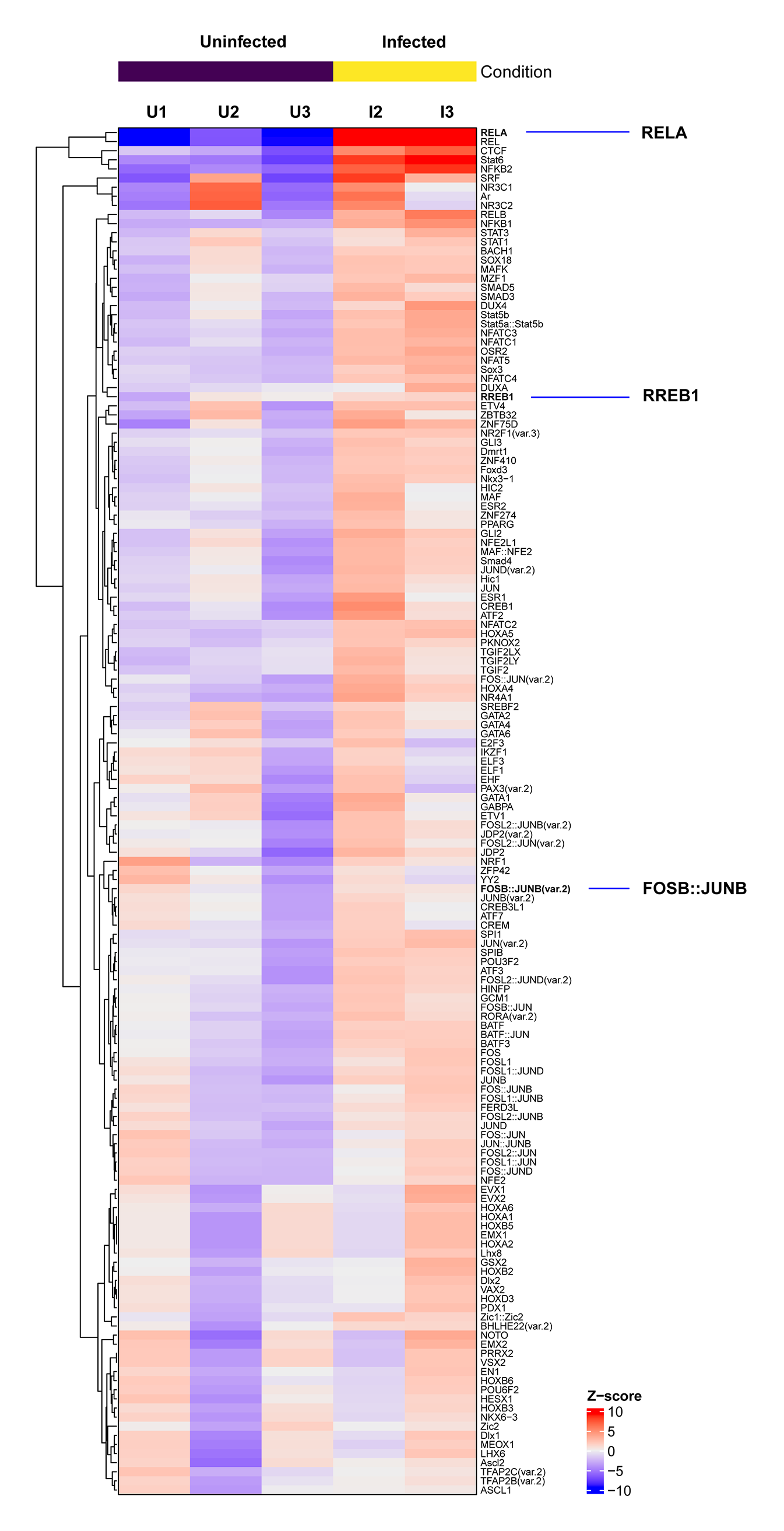

Supplement: S4 Fig — We see the RELA, RREB1 and FOSB::JUNB motifs now accompanied by many other transcription factor motifs, including many NFκB family members and AP-1 targets. These are all listed in S3 Table. (TIF) [file pone.0275226.s004.tif]

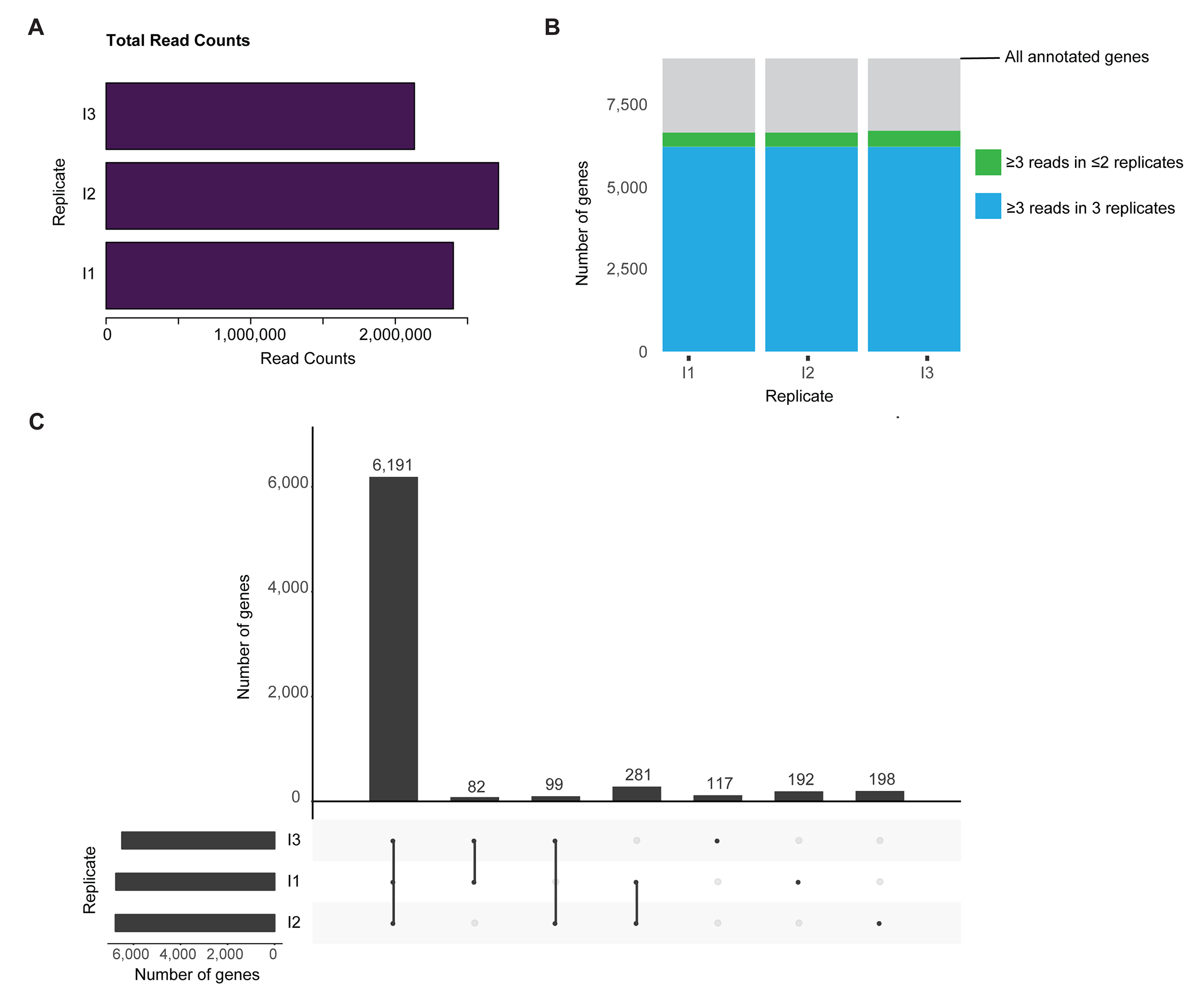

Supplement: S5 Fig — In (A) we show the number of RNA-seq reads from T. gondii to be consistently greater than 2 million in all replicates. In (B) we illustrate how most genes in the T. gondii genome are represented consistently across replicates, also showing this quantitatively in the UpSet plot of panel (C). (TIF) [file pone.0275226.s005.tif]

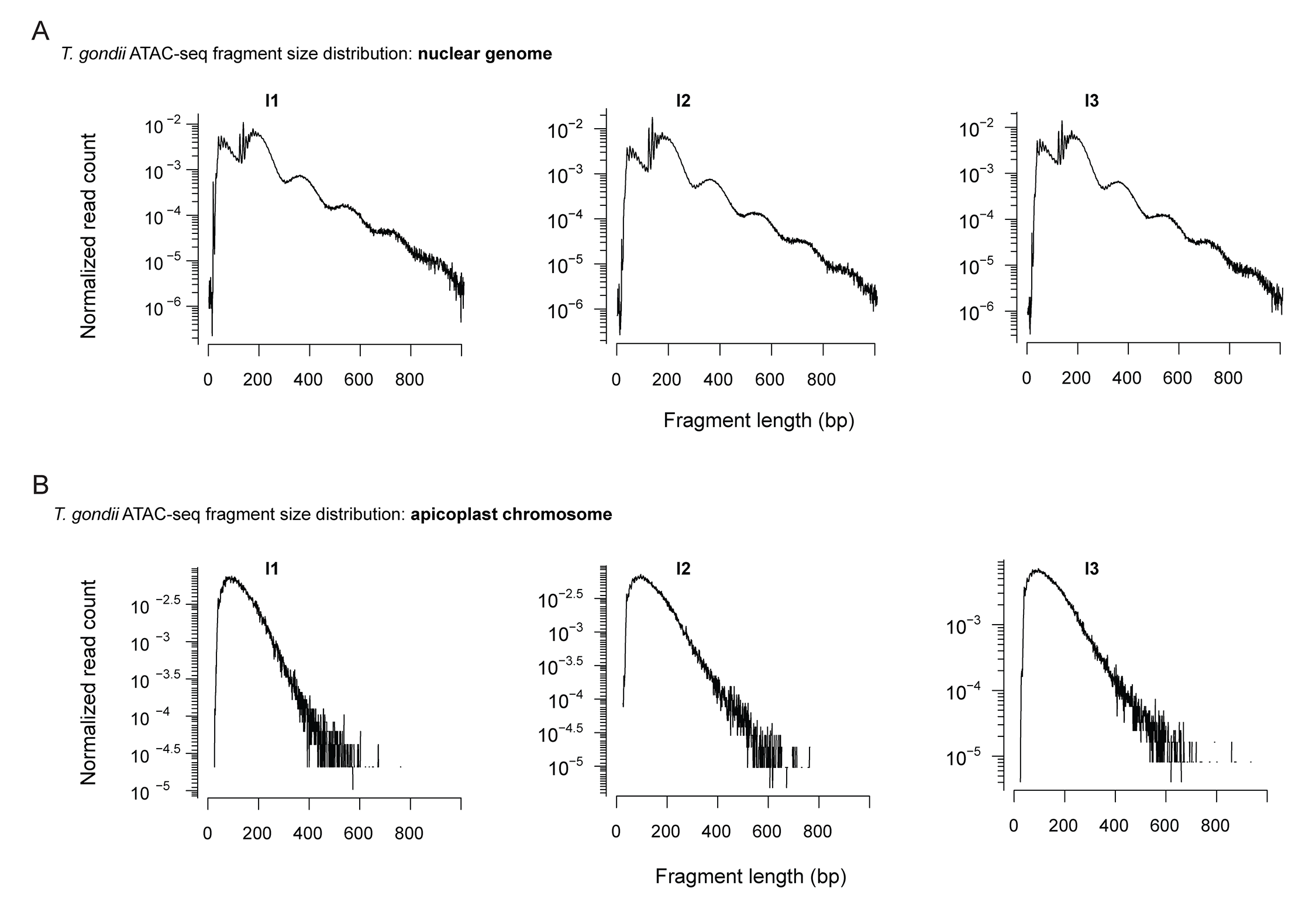

Supplement: S6 Fig — The expected nucleosomal periodicity pattern of chromatin is revealed from the insert size plots of panel (A), representing the reads aligning to the T. gondii nuclear genome, whereas in (B) we see no evidence for such nucleosomal organization in reads mapping to the T. gondii apicoplast DNA. (TIF) [file pone.0275226.s006.tif]

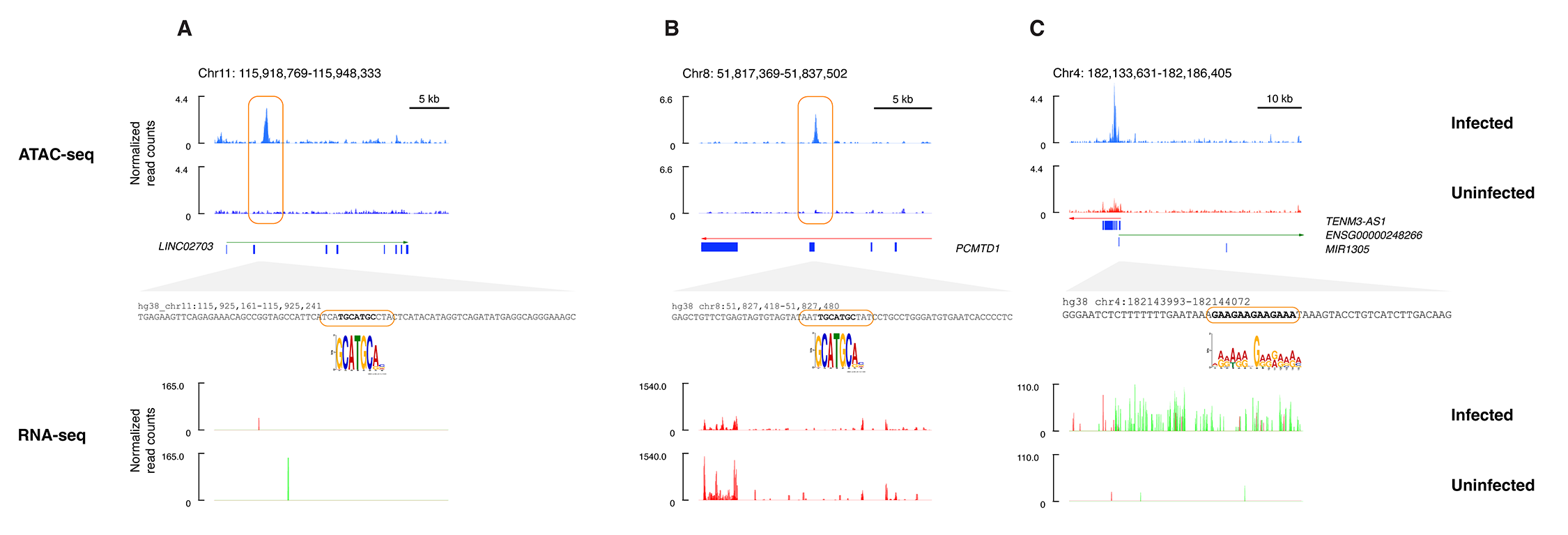

Supplement: S7 Fig — (TIF) [file pone.0275226.s007.tif]
